# Effect of a quality improvement intervention for management of preterm births on outcomes of all births in Kenya and Uganda: A secondary analysis from a facility-based cluster randomized trial

**DOI:** 10.7189/jogh.12.04073

**Published:** 2022-12-29

**Authors:** Rakesh Ghosh, Phelgona Otieno, Elizabeth Butrick, Nicole Santos, Peter Waiswa, Dilys Walker, Preterm Birth Initiative Kenya and Uganda Implementation Research Collaborative

**Affiliations:** 1 University of California, San Francisco, Institute for Global Health Sciences, USA; 2 Center for Clinical Research, Kenya Medical Research Institute, Nairobi, Kenya; 3 Makerere University, School of Public Health, Uganda; 4 Department of Global Public Health, Karolinska Institutet, Sweden; 5 University of California, San Francisco, School of Medicine, Department of OB/GYN and Reproductive Sciences, USA

## Abstract

**Background:**

A large proportion of early neonatal deaths occur at the time or on the first day of birth. The Preterm Birth Initiative East Africa (PTBi EA) set out to decrease mortality among preterm births through improving quality of facility-based intrapartum care. The PTBi EA cluster randomized trial’s primary analysis showed the package reduced intrapartum stillbirth and neonatal death among preterm infants. This secondary analysis examines the impact of the PTBi intervention package on stillbirth and predischarge newborn deaths combined, among all births in 20 participating facilities in Kenya and Uganda.

**Methods:**

Eligible facilities were pair-matched and randomly assigned (1:1) into either the intervention or the control group. All facilities received support for data strengthening and a modified World Health Organization (WHO) Safe Childbirth Checklist; facilities in the intervention group additionally received provider mentoring using PRONTO simulation and team training as well as quality improvement collaboratives. We abstracted data from maternity registers.

**Results:**

Of the total 29 442 births that were included, Kenya had 8468 and 6465 births and Uganda had 8719 and 5790 births, in the control and intervention arms, respectively. There were 935 stillbirths and predischarge newborn deaths in the control arm and 439 in the intervention arm. The adjusted odds ratio (aOR) for the effect of the intervention on the combined outcome, among all births, was 0.96 (95% confidence interval (CI) = 0.69-1.32), which was different by country: Kenya – 1.12 (95% CI = 0.72-1.73); Uganda – 0.65 (95% CI = 0.44-0.98); *P*_interaction_  = 0.025. These trends were similar after excluding the PTBi primary cohort.

**Conclusions:**

The intervention package improved survival among all births in Uganda but not in Kenya. These results suggest the importance of context and facility differences that were observed between the two countries.

**Registration:**

This trial is registered with ClinicalTrials.gov, NCT03112018.

Globally, an estimated 2.7 million neonatal deaths occur annually, of which preterm birth (PTB: birth before 37 completed weeks of gestation) is the leading cause and the majority occur in low- and middle-income countries (LMIC) [1]. Another 2.6 million pregnancies end in stillbirth, of which more than three-quarters occur in South Asia and sub-Saharan Africa, combined [2]. In 2017, almost 300 000 women died from pregnancy-related causes, 86% of which occurred in sub-Saharan Africa and South Asia [3]. About 40% of the neonatal deaths and stillbirths and 46% of the maternal deaths occur around the time of labor or on the day of birth [4]. These statistics highlight the importance of quality intrapartum care and its significance to maternal and newborn survival.

The Preterm Birth Initiative – East Africa (PTBi EA) set out to decrease the burden of mortality among PTB through improving quality of facility-based intrapartum care. Per our study logic model of change [5], an intrapartum-immediate newborn quality improvement package focused on preterm identification and management would result in improved survival. While the PTBi EA intervention package emphasized key topics related to PTB, it also sought to improve overall quality of care during the intrapartum and immediate postnatal period in the facility, which research has shown to be key to saving newborn lives [6]. Specifically, the intervention package was developed based on available evidence that up to two-thirds of neonatal deaths could be averted by improved uptake of evidence-based practices described by the Every Newborn Action Plan [4]. Previous research recommended the use of priority packages in an integrated approach for effective improvement in neonatal outcomes [4,6]. Each of the PTBi package components was chosen because it met stakeholder requirements of: (a) having the potential to improve quality of care for preterm infants as well as for the mother-baby dyad more generally, and (b) demonstrable effectiveness as standalone interventions based on prior evidence. Our intervention package reinforced evidence-based practices, many of which should be observed for all births including those of intrapartum monitoring and neonatal resuscitation [7].

The PTBi EA trial’s primary analysis showed the package to be effective in reducing intrapartum stillbirth and neonatal death (primary outcome), as well as predischarge newborn mortality (secondary outcome) among preterm infants [8]. Because stakeholders involved in the design and implementation of the intervention package prioritized elements believed to improve care for all births, we hypothesized that it would also impact survival among all births. Therefore, the objective of this secondary analysis was to examine the impact of the PTBi quality improvement intervention package on stillbirth and predischarge newborn mortality, as a composite outcome, among all births in participating facilities starting in October 2016 up to June 2018 in Uganda and up to March 2019 in Kenya. As the facilities in the two countries were heterogenous by several accounts, we also examined if the intervention effect was differential by country. We additionally investigated the impact of the intervention on predischarge maternal mortality. Our manuscript follows the recommendations of the CONSORT Statement [9] that is relevant for cluster randomized trials.

## METHODS

### Study design and setting

As described elsewhere, PTBi EA conducted an unblinded, pair-matched cluster-randomized controlled trial (cRCT) in select health facilities in Migori County, Kenya and Busoga Region, Uganda [5]. Interventions were delivered at the facility level. Twenty-three rural and peri-urban facilities were assessed for inclusion in the cRCT (Figure S1 in the **Online Supplementary Document**) with the following criteria: (a) 24-hour labor and delivery services, (b) at least 200 births per year, and (c) a comparable facility in the same country to pair-match. Three facilities - one county referral hospital in Kenya, one district referral and one regional referral hospital in Uganda - were excluded from the cRCT because of lack of comparable facilities to pair-match. However, these referral facilities received the intervention package in order to benefit from any quality improvement activities, achieve and maintain similar quality of care standards, and to prevent lower-level facilities from referring to a center with inferior quality of care.

In Kenya, the cRCT included 14 public and two non-profit missionary facilities. Among these, two performed caesarean sections and none had functional newborn care units or onsite pediatricians. One control site added caesarean section capacity while the study was ongoing. Together, the 16 facilities had approximately 11 000 deliveries per year, during the study period. On average, one to two midwives covered each shift.

In Uganda, the cRCT included four district-level facilities, two public and two non-profit missionary hospitals. All four facilities did caesarean sections, and each had a newborn care unit without capacity for continuous positive airway pressure or an onsite pediatrician. Together, the four facilities had approximately 9000 deliveries per year, during the study period. The facilities in Uganda were higher volume than those in Kenya, with two to three midwives per shift.

### Matching and randomization

Counts of indicators between June 2015 and May 2016 from maternity registers were used to match and generate 10 facility pairs. A non-bipartite matching algorithm was used to match by country, monthly deliveries, deliveries to staff ratio, stillbirth rate, low-birthweight rate, and predischarge newborn death rate [5,8]. Field teams reviewed the preliminary matched pairs, and five of the ten pairs were re-matched on the basis of local knowledge of functional level and facility type. After final matching, a study statistician randomly assigned one from each pair to the intervention group. Nature of the intervention rendered allocation concealment impossible.

### Intervention

The intervention package for the trial was designed using an integrated approach to optimize care for the mother-baby dyad, to impact provider behavior at critical moments during triage, labor, birth, and the early neonatal period. The four components of the interventions package are described in detail elsewhere [7]. Additionally, a summary is provided in Figure S2. in the **Online Supplementary Document**. The intervention package included: (a) PRONTO simulation and team training; (b) quality improvement collaboratives; (c) modified Safe Childbirth Checklist (mSCC); and (d) data strengthening [5,7]. The package aimed to strengthen provider skills, teamwork and emphasized use of evidence-based practices for PTB, including but not limited to the appropriate administration of antenatal corticosteroids, immediate skin to skin contact, breastfeeding, newborn resuscitation, and feeding preterm neonates. PRONTO training focused on provider skill development in emergency obstetric and neonatal care, including preeclampsia, hemorrhage and a focus on PTB, while quality improvement collaboratives allowed intervention facilities to discuss quality improvement projects and their effects on target indicators. The mSCC served to provide reminders of key evidence-based practices, with an emphasis on PTB and triage processes prior to admission. The data strengthening component focused on maternity register completion and data quality particularly for accurate gestational age and outcome indicators. Data strengthening and introduction of the mSCC were implemented in both intervention and control facilities beginning in May 2016 and continuing throughout the study. In October 2016, PRONTO training and quality improvement collaboratives began in the 10 intervention facilities and continued throughout the study period. Before the trial began, sites were assessed and if basic equipment and/or supplies, such as functional digital scales and neonatal bag and masks, were found unavailable, PTBi EA provided these items to standardize resource availability across sites. These expenses did not exceed US$50 000 in either country.

### Data collection

Anonymized individual-level data for all births were extracted each month from facility-based maternity registers by PTBi study staff and entered into an encrypted Open Data Kit platform. Each mother and infant were assigned unique but linked identifiers. Data collected include, maternal (age, multiplicity, referral status) and newborn (birth weight, gestational age at birth, APGAR scores, infant sex) characteristics, mode of delivery and maternal and newborn status at birth and discharge.

While preterm neonates (defined as fresh stillbirths and livebirths weighing 1000-2499 g irrespective of gestational age, or 2500-2999 g with a recorded gestational age of less than 37 weeks) were invited to participate for follow-up at 28 days after birth for the cRCT primary analysis, there was no provision for follow-up after discharge for the remaining births in the cohort. For our primary cRCT analysis, any stillborn and liveborn neonates weighing less than 1000 g, irrespective of gestational age, were excluded because they were considered previable in both countries. However, all births registered in the maternity ward, regardless of their birth weight or gestational age, were included in this secondary analysis.

### Outcomes

Birth outcome was determined by triangulating delivery status, APGAR scores and baby discharge status. Stillbirths included intrapartum stillbirth (recorded as “fresh stillbirth” and defined as intrauterine death of a fetus during labor or delivery), antepartum stillbirth (recorded as “macerated stillbirth” and defined as intrauterine death of a fetus before the onset of labor showing degenerative changes), and those recorded only as “stillbirth” in the maternity register. Sometimes the delivery status and/or the baby discharge status recorded only stillbirth, ie, a sub type was not defined, and the APGAR scores were zero. Those cases were designated as “undefined stillbirth.” To reconcile the heterogeneity, we included intrapartum, antepartum and undefined stillbirths into one overall group as “stillbirth.” Predischarge newborn mortality was defined as death of a liveborn baby before facility discharge, which we combined with stillbirth and investigated as a composite outcome in this secondary analysis. Pregnancy-related maternal death was defined as deaths from any cause related to or aggravated by pregnancy or its management during pregnancy and childbirth, as recorded in the maternity register.

### Sample size

**Figure 1:**
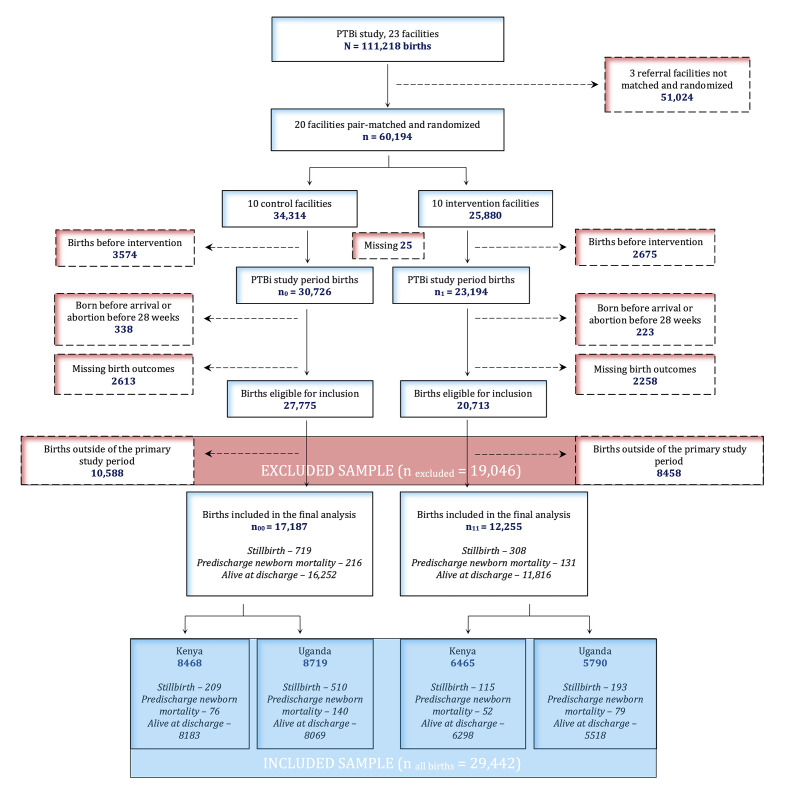
The Preterm Birth Initiative (PTBi) study trial profile for all births. The included and the excluded sample were compared to examine selection bias.

Sample size for the primary outcome of the PTBi cRCT is presented elsewhere [5,8]. For this secondary analysis with 29 442 births (**Table 1** and **Figure 1**) and estimated intracluster correlation of 0.02, the power is 56% at the 5% significance level to detect a 1.8% difference between the two arms (5.4% vs 3.6%, **Table 2**), for the composite outcome. For Uganda, with 14 509 births, this study had 78% power to detect a 2.8% (7.5% vs 4.7%, **Table 2**) difference between the study arms.

**Table 1:** Characteristics (%, n) of all births in the two countries, aggregated and separately*

Characteristics	Both countries aggregate				Kenya				Uganda			
	Control (n = 17 187)		Intervention (n = 12 255)		Control (n = 8468)		Intervention (n = 6465)		Control (8719)		Intervention (5790)	
Maternal characteristics	%	n	%	N	%	n	%	n	%	n	%	n
Age categories (years)												
13-17	7.6	1297	8.9	1085	8.3	702	11.5	744	6.9	595	6.0	341
18-35	87.0	14 860	86.2	10 488	87.1	7348	84.2	5427	87.0	7512	88.5	5061
36-53	5.4	919	4.9	601	4.6	391	4.3	274	6.1	528	5.7	327
Caesarean sections	20.7	3499	14.1	1724	11.4	957	3.8	247	29.8	2542	25.7	1477
Vaginal delivery	79.9	13 812	86.2	10 640	88.6	7461	96.2	6186	70.2	5988	74.4	4288
Multiple gestations	4.5	780	3.9	475	3.8	325	2.7	173	5.2	455	5.2	302
Singletons	95.5	16 407	96.1	11 780	96.2	8143	97.3	6292	94.8	8264	94.8	5488
Neonatal characteristics												
Male	50.4	8661	50.8	6228	49.3	4171	51.7	3340	51.5	4490	49.9	2888
Birth weight <2500 g	7.9	1347	8.9	1067	5.4	454	6.5	418	10.5	893	11.6	649
Gestational age <37completed weeks	10.9	1872	12.6	1541	9.5	800	10.3	667	12.3	1072	15.1	874

*Numbers will not add up to the total in each study arm because of missing data.

**Table 2:** Effect of PTBi intervention (odds ratio, OR) on neonatal and maternal outcomes in all births across the two countries, aggregated and separately*

	Both countries aggregate				Kenya			Uganda		
	Control	Intervention	OR (95% CI)	Interaction *P*-value	Control	Intervention	OR (95% CI)	Control	Intervention	OR (95% CI)
Stillbirth + predischarge newborn mortality (combined)	935/17 187	439/12 255	0.96 (0.69-1.32)	0.025†	285/8468	167/6465	1.12 (0.72-1.73)	650/8719	272/5790	0.65 (0.44-0.98)
Stillbirth	719/17 187	308/12 255	0.88 (0.65-1.20)	0.029†	209/8468	115/6465	1.04 (0.79-1.36)	510/8719	193/5790	0.59 (0.36-0.97)
Predischarge newborn mortality	216/16 468	131/11 947	0.96 (0.83-1.10)	0.098	76/8259	52/6350	1.12 (0.86-1.47)	140/8209	79/5597	0.88 (0.76-1.02)
Predischarge maternal mortality	29/15 294	16/10 492	0.89 (0.52-1.51)	0.939	9/7076	5/5521	0.86 (0.30-2.43)‡	20/8218	11/4971	0.90 (0.45-1.80)

CI – confidence interval, PTBi – preterm birth initiative

*Models are adjusted for matched pairing of facilities and clustering of births within facilities.

†Interaction *P*-value for Country × Intervention, suggesting statistically significant difference in the outcome between the two countries.

‡Two pairs of clusters (6 facilities) were dropped from the Kenya maternal mortality model because there were no maternal deaths in either the control or the intervention facilities. These results are likely unstable because of few outcomes.

### Statistical analysis

For this secondary analysis, all births (*n _all births_* = 22 442) were included (**Figure 1**) and their characteristics were compared across arms to examine arm balance. We also examined the trend of the composite outcome stillbirth and predischarge newborn mortality, by arms, over the study period. We followed similar analytical methods to measure the intervention effect, as reported previously for the primary analysis [8]. With individual births as unit, we performed intention-to-treat analysis using multilevel logistic regression adjusting for pairing because of the pair-matched design. We used a random intercept for each facility, accounting for clustering of deliveries within facilities and quantified appropriate standard errors. We quantified the overall effect of the PTBi intervention and the differential effect by country, as the facilities in the two countries were relatively heterogenous. The statistical significance of the differential effect (interaction) by country was examined using a product term in the model, following which we fit stratified models. The main results report the effect of intervention on the composite outcome as well as the other outcomes by comparing all births between the intervention and the control facilities, during the study period.

We have previously reported an effect of the PTBi intervention on the primary outcome that included only preterm and low birth weight neonates followed up to 28 days (*n _PTBi primary cohort_* = 2938) [8]. In order to understand if any effect observed between the intervention and outcomes among all births is independent of the effect observed in the PTBi primary cohort reported previously, we conducted sensitivity analysis_1 by excluding the PTBi primary cohort (ie, *n _all births_ – n _PTBi primary cohort_* = 27 397). Further, in sensitivity analysis_2 we excluded all preterm neonates to examine if any observed effect was driven by the effect on PTB. In other words, the sensitivity analysis_2 was restricted to term births (ie, *n _all births_ – n _all preterm_* = 25 003). This sensitivity analysis excluded the PTBi primary cohort, preterm macerated stillbirths that were ineligible for the PTBi primary cohort, mothers who delivered preterm and declined consent for 28-day follow-up after birth, and mothers who were not consented for other reasons. Finally, we examined selection bias by comparing study characteristics of the included (*n _all births_* = 29 442) with the excluded sample (*n _excluded_* = 19 046), as shown in **Figure 1**. To understand if differential selection of the study sample could be a potential alternative explanation for any observed effect, we performed sensitivity analysis_3 by combining the included (*n _all births_*) with the excluded births (*n _excluded_*). All hypothesis tests were two-tailed at the 5% significance level. Analyses were conducted using STATA version 17.0 (StataCorp).

The cRCT was approved by the respective Review Boards at the University of California San Francisco (16–19162, 29-March-2016), the Kenya Medical Research Institute Scientific (KEMRI/SERU/CCR/0034/3251, 12-May-2016), the Makerere University Higher Degrees and the Uganda National Council of Science and Technology (MUSPH HDREC 395, 17-June-2016). We obtained a waiver of consent to obtain de-identified line-by-line data from maternity registers. The trial is registered on ClinicalTrials.gov, NCT03112018.

## RESULTS

The number of participants in the two study arms and the exclusions are shown in **Figure 1**. After all exclusions, 29 442 births were included, with 17 187 in the control arm and 12 255 in the intervention arm. Kenya had 8468 and 6465 and Uganda had 8719 and 5790 births in the control and intervention arms, respectively.

Across both countries aggregated, about 87% of all births were among women aged between 18 and 35 years evenly distributed across the arms (**Table 1**). About 21% had cesarean section in the control arm compared to 14% in the intervention arm. Likewise, multiple births were slightly higher in the control (4.5%) than in the intervention arm (3.9%). In contrast, the proportions of low birth weight and PTB were generally lower in the control arm than in the intervention arm.

By country, Kenya had a higher proportion of births among women less than 18 years and a much lower proportion of cesarean section compared to Uganda (**Table 1**). Multiple births were lower in Kenya than in Uganda. Proportions of neonates with low birth weight and PTB were generally lower in Kenya than in Uganda (**Table 1**).

**Figure 2:**
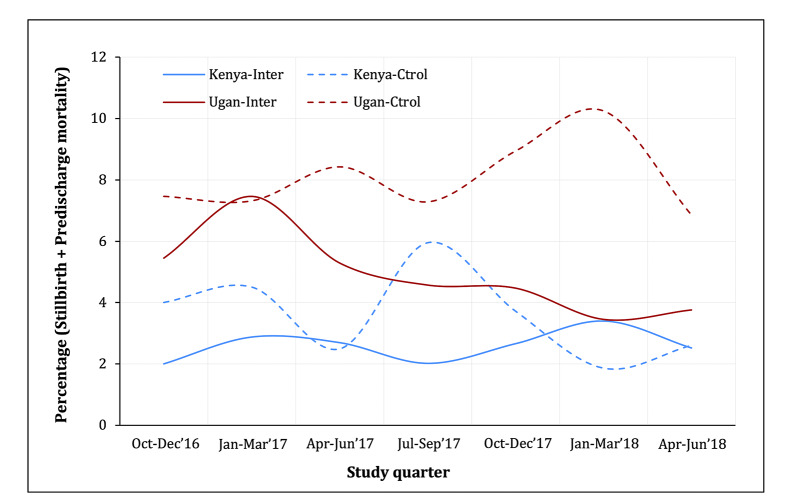
Temporal trends of the combined percentage of stillbirths and predischarge newborn mortality among all births, by the Preterm Birth Initiative (PTBi) study trial arms in Kenya and Uganda, reported for each quarter. In Kenya, we truncated in June 2018 to show comparable time points for both countries in this graph.

Over the study period and aggregated across both countries, there were 935 stillbirths and predischarge newborn mortality in the control and 439 in the intervention arm, with rates of 54.4 and 35.8 per 1000 total births, respectively. This rate was lower in Kenya (33.7 controls and 25.8 intervention) than in Uganda (74.5 controls and 47.0 intervention). The adjusted OR for the effect of the intervention on the composite outcome was 0.96 (95% CI = 0.69-1.32), which was different by country: Kenya – 1.12 (95% CI = 0.72-1.73); Uganda – 0.65 (95% CI = 0.44-0.98); *P*_interaction_ = 0.025 (**Table 2**). The temporal trends in the composite outcome show a difference by arm in Uganda but not for Kenya (**Figure 2**). The results for stillbirths only were similar to the composite outcome (**Table 2**) with an OR of 0.88 (95% CI = 0.65-1.20) aggregated for both countries, 1.04 (95% CI = 0.79-1.36) in Kenya, and 0.59 (95% CI = 0.36-0.97) in Uganda. However, predischarge newborn mortality and predischarge maternal mortality were similar in both arms and there was no statistically significant effect of the intervention on these two outcomes, overall or by country (**Table 2**).

In sensitivity analysis_1 (**Table 3**), when the PTBi primary cohort was excluded, the results remained largely the same as in all births (aggregated for both countries – 0.93 (95% CI = 0.68-1.28); Kenya – 1.07 (95% CI = 0.84-1.35); Uganda – 0.62 (95% CI = 0.41-0.96); *P*_interaction_ = 0.018,). In sensitivity analysis_2 (Table S1 in the **Online Supplementary Document**), when we restricted to term births, the results became stronger (aggregated for both countries – 0.77 (95% CI = 0.56-1.05); Kenya – 0.99 (95% CI = 0.77-1.29); Uganda – 0.59 (95% CI = 0.45-0.79); *P*_interaction_ = 0.006). Furthermore, compared to the results among all births, the intervention effect for predischarge newborn mortality became significant, aggregated for both countries (0.83, 95% CI = 0.70-0.97) and for Uganda (0.77, 95% CI = 0.75-0.79). The effect on predischarge maternal mortality, which was based on few events, flipped direction and remained statistically non-significant.

**Table 3:** Effect of PTBi intervention (odds ratio, OR) on neonatal and maternal outcomes in all births across the two countries, aggregated and separately, after excluding the PTBi primary cohort*

	Both countries aggregate				Kenya			Uganda		
	Control	Intervention	OR (95% CI)	Interaction *P*-value	Control	Intervention	OR (95% CI)	Control	Intervention	OR (95% CI)
Stillbirth + predischarge neonatal mortality (combined)	773/16 113	348/11 284	0.93 (0.68-1.28)	0.018†	240/8032	135/6016	1.07 (0.84-1.35)	533/8081	213/5268	0.62 (0.41-0.96)
Stillbirth	611/16 113	252/11 284	0.83 (0.63-1.09)	0.023†	181/8032	95/6016	1.02 (0.81-1.29)	430/8081	157/5268	0.56 (0.33-0.95)
Predischarge neonatal mortality	162/15 502	96/11 032	0.97 (0.81-1.14)	0.101	59/7851	40/5921	1.18 (0.83-1.67)	103/7651	56/5111	0.86 (0.72-1.02)
Predischarge maternal mortality‡	19/13 907	15/9116	1.28 (0.82-2.00)	0.607	6/6296	4/4598	1.05 (0.39-2.79)	13/7611	11/4518	1.39 (0.85-2.27)

CI – confidence interval, PTBi – preterm birth initiative

*Models are adjusted for matched pairing of facilities and clustering of births within facilities.

†Interaction *P*-value for Country × Intervention, suggesting statistically significant difference in the outcome between the two countries.

‡Three pairs of clusters (6 facilities) were dropped from the maternal mortality model because there were no maternal deaths in either the control or the intervention facilities. The results for Kenya are likely unstable because of few outcomes.

We observed a difference in distribution of stillbirths by study arms between all births and the excluded births (Table S2 in the **Online Supplementary Document**). However, in sensitivity analysis_3, when all births were combined with excluded births (*n _all births_* : 29 442 + *n _excluded_* : 19 046 = 48 488, **Figure 1**), the effect of the intervention and the difference by country remained similar (Table S3 in the **Online Supplementary Document**).

## DISCUSSION

The PTBi intervention package did not show a measurable impact on newborn survival among all births in the 20 hospitals across Kenya and Uganda combined. However, results suggest improved newborn survival only in the Ugandan hospitals demonstrating that this package of facility-based interventions focused on PTB, has the potential to improve survival of all births in the facility. The environment of continuous training and quality improvement likely increases skills among providers, but also serves to refresh overall evidence-based care for all births and affects general quality of intrapartum care in the facilities. For predischarge newborn mortality alone or for predischarge maternal mortality, the intervention did not show significant effects overall or by country, though the confidence interval for the former is more in support of an effect, rather than null effect [10]. In the case of predischarge maternal mortality, no significant effect is likely due to too few events. Robustness of the results to various sensitivity analyses reaffirms that these are likely true effects and not potentially driven by alternative explanations.

The lack of significant results in Kenya suggests that the context of the two countries may be key as an important implementation research finding. Kenya had 16 facilities ranging from health centers to sub-county hospitals, reflecting more variation in the types of cases attended, availability of medical doctors, infrastructure and equipment across facilities. For example, only 3 of the 16 Kenyan facilities, while all 4 Ugandan facilities, had caesarean sctions capacity and newborn units. Furthermore, when counts of births and outcomes were compared within matched pairs, the results were more heterogeneous in Kenya than in Uganda. Kenya also experienced a protracted health worker strike which may have caused degradation in provider skills (including possible degradation in the effect of the intervention) and accelerated staff turnover. On the other hand, all 4 Ugandan hospitals were at the district level and staffed by on-site pediatricians and obstetricians. Stronger results for Uganda could also be due to a number of factors including greater improvement in care, given worse rates to begin with, better fit between the intervention package and the levels of care provided in these facilities, and/or factors affecting fidelity of implementation. For example, with larger delivery volumes in Uganda facilities than Kenya facilities, this may have provided more frequent opportunity for providers to implement skills learned through the PTBi intervention package. These observations illustrate the importance of recognizing and considering contextual factors that may influence both intervention implementation and outcomes.

One possible implication of these results is that packages such as the PTBi may be optimally effective at a certain level of the health care system. Facilities in Kenya were much smaller and may not have been optimally organized to most benefit from the intervention, or high staff turnover may have had a disproportionate impact. The same intensity of intervention may have impacted more in lowering the rates in Uganda (because the rates were much higher to begin with) than in Kenya by better catering to the health system needs in that country [11]. Thus, a range of factors may have contributed towards marked improvement in Uganda but not in Kenya.

As this analysis investigates a secondary outcome, and in light of no overall effect, the results should be viewed critically. This group previously reported significant effect of the intervention on the survival of preterm infants in the intervention facilities compared to the control facilities [8]. This article is a logical extension of the analysis to all (preterm and term) births. The remarkably similar magnitude of the intervention effect among all births in Uganda (OR = 0.65, 95% CI = 0.44-0.98, n ~ 14 500) reported here with that reported previously among PTB births in Kenya and Uganda (OR = 0.66, 95% CI = 0.54-0.81, n ~ 3000) is unlikely to be by chance. We have demonstrated that the intervention effect among all births is not driven by the effect on PTB (sensitivity analysis_1). Rather, the results hold when the PTBi primary cohort and all other PTB were excluded, demonstrating that the effect was more likely to be on term births as well as PTB. Furthermore, in term births (sensitivity analysis_2), the intervention effect was significant for predischarge newborn mortality, overall as well as in Uganda, unlike in all births. The significance achieved despite a smaller sample in sensitivity analysis_2 was possible because the effect was larger in magnitude in term births than in all births. This finding signifies that the intervention increased chances of survival among the term births as it did among the PTB (reported earlier [8]). This interpretation is further supported by results obtained after adjusting for APGAR score when the intervention effect becomes non-significant (Table S4 in the **Online Supplementary Document**). We hypothesize that the intervention effect can be explained by APGAR at 5 minutes since it is in the causal pathway between intervention and increased survival (as a mediator). The intervention likely improved providers’ ability to assess newborn status at birth as well as the confidence to manage a neonate not spontaneously breathing at birth. In fact, the intervention included reinforcement of key actions providers should take in the first 5 minutes of life for a newborn not spontaneously breathing at birth (eg, immediate neonatal resuscitation).

We explored potential alternative explanations for the intervention effect. The series of exclusions (**Figure 1**) leading to the final study sample could have induced selection bias. More stillbirths were excluded in the intervention arm than in the control arm (Table S2 in the **Online Supplementary Document**). However, this was not the case for predischarge newborn mortality. Large differences between the included and excluded births were not observed for any other characteristics (Table S2 in the **Online Supplementary Document**). We conducted sensitivity analysis to examine if the main results were due to differential selection of study samples in the two arms. Results presented in Table S3 in the **Online Supplementary Document** demonstrate that selection bias, if any, is unlikely to explain the intervention effect. Given the nature of the intervention and assessments of outcomes, which are expected to be uniform within a facility, measurement bias is also unlikely.

Our findings are similar to others taking a package approach including quality improvement and provider training. Ashish et al. reported a stepped wedge trial in Nepal that included both quality improvement interventions and provider training focused on neonatal resuscitation in public hospitals with at least 1000 births per year [12]. Of note, our facilities in Uganda all had more that 1000 births per year, whereas Kenyan facilities ranged from 310-1599 deliveries per year [8]. Similar to our results, the Nepal study found a positive impact on stillbirth and newborn mortality, though there was some variability across facilities [12]. In addition, a systematic review of quality improvement as a single intervention was reported to have variable effects, while quality improvement combined with provider training shows positive impact on health outcomes [13]. Unique components of the PTBi intervention like simulation and team training implemented in Mexico demonstrated that it can reduce neonatal mortality [14]. In other studies, from Guatemala and India, PRONTO’s simulation and team training increased use of evidence-based practices in normal and complicated deliveries when these activities were integrated along with didactic teaching and demonstrations [15-18]. Similar packaged interventions are currently being implemented in several sub-Saharan countries to examine if individual components can synergistically act with each other to bring about a larger reduction in perinatal mortality [19,20].

The study has several strengths as well as limitations. Amongst the key strengths are the randomized design and the availability of data on all births that allowed investigation beyond PTB. The consistency and the logical coherence of these results with the earlier results on PTB lends credibility. This trial is an important addition to the literature in its demonstration that a package approach can be advantageous, though it may not be equally effective in all contexts, pointing to the need for further implementation research in this area. An important limitation was power, despite which the Uganda-specific results were strong enough to reach statistical significance. The intervention was a package of four components (quality improvement collaboratives, PRONTO simulation and team training, mSCC and data strengthening), two of which (mSCC and data strengthening) were implemented in the control facilities. Thus, the comparison group in this trial were not pure controls, which could potentially underestimate the effect. There was a health worker strike in Kenya during the trial, which likely affected provider turnover, provider motivation, and retention of any pre-strike learning. Data for the study were obtained from facility registers, which may have quality issues, especially during excessive delivery loads. However, data strengthening component of the intervention likely mitigated some of its effect, as described elsewhere [8]. There may have been some misclassification between stillbirth and predischarge newborn mortality among infants who died very shortly after birth. Such misclassification will not affect the results for the combined outcome but may have affected the stillbirths or predischarge mortality specific results. Finally, despite best efforts, perfect matching of facilities within pairs may not have been achieved, especially regarding immoveable infrastructure and community characteristics to which these facilities cater. Such differences could have left residual imbalance between arms.

## CONCLUSIONS

The PTBi intervention appears to have improved survival not only among PTB, as reported earlier, but also among all births in Uganda. We did not observe the same effect in Kenya, which is intriguing and hints towards factors that were contextually different in the two countries. Efforts to sustain the achieved improvement should be the next logical step of focus. From the research perspective, it will be important to delineate the differences in package implementation, settings and care that might have led to these results.

## Additional material:


Online Supplementary Document
