# Supplementary material for: Effect of a quality improvement intervention for management of preterm births on outcomes of all births in Kenya and Uganda: A secondary analysis from a facility-based cluster randomized trial
Source: J Glob Health. 2022 Dec 29;12:04073. doi: 10.7189/jogh.12.04073 (PMC9799078; doi:10.7189/jogh.12.04073)
Supplement: Online Supplementary Document [file jogh-12-04073-s001.pdf]

Effect of a quality improvement intervention for management of preterm births on outcomes of all births in Kenya and Uganda: a secondary analysis from a facility-based cluster randomized trial

Rakesh Ghosh, Phelgona Otieno, Elizabeth Butrick, Nicole Santos, Peter Waiswa, Dilys Walker and the Preterm Birth Initiative Kenya and Uganda Implementation Research Collaborative

Figure S1. PTBi study timeline demarcating the baseline period, study period, strike period and the time when the control facilities received the intervention.

Facility name	***Country***	***I/C/R****	***2016***			***2017***					***2018***		***2019***
			Mar – Sep	*Oct – Nov*	*Dec*	*Jan*	*Feb*	*Mar – May*	*Jun – Nov*	*Dec*	*Jan – Jul*	*Aug – Dec*	*Jan – Mar*
Sub-DH 1		C											
Sub-DH 2		C											
DH 1		C			Strike		Strike						
HC 1		C							Strike				
Sub-DH 3		C											
Sub-DH 4		C											
Mission 1		C											
HC 2		C											
HC 3	Kenya	I	Baseline										
DH 2		I			Strike		Strike						
Sub-DH 5		I							Strike				
HC 4		I											
DH 3		I											
Mission 2		I											
HC 5		I											
Sub-DH 6		I			Strike		Strike		Strike				
RH 1		R											

Mission	3	Uganda	C			Received lighter intervention
HC	6		C			
HC	7		I			Received lighter intervention
Mission	4		I			
RH	2		R			
RH	3		R			

**I – Intervention, C – Control or R – Referral facility*

# – DH: District Hospital; Mission: Mission Hospital; HC: Health Center; RH: Regional Hospital

Note – Empty regions represent control period, blue regions represent intervention period, purple regions represent sustained interventions post trial data collection in Uganda and the grey regions represent the strike in the facilities during the study.

Figure S2. A detailed description of components of the Preterm Birth Initiative – East Africa pair matched cluster randomized trail interventions package. Adapted from an earlier publication (https://doi.org/10.1016/S2214-109X(20)30232-1) that has a CC BY 4.0 license.

Data strengthening

- Annual workshops to review indicator definitions and standardisation, eg, gestation, birthweight, 1-min and 5-min Apgar scores, birth and discharge status
- Provision of pregnancy wheels and tape measures to improve gestational age assessment
- Best practice recommendations for chart room organisation and clinical chart filing systems
- Monthly site visits to collect birth register data and review data completeness and correctness
- Creation of a Data Dashboard with provision of site-specific monthly reports
- Bi-annual data quality assessments and findings dissemination with facility stakeholders

Target personnel: health records officers and staff, maternity ward and newborn care providers
Frequency: 1–2 h per month per facility (about 20 h per year)
Fidelity: two data quality assessments in Uganda (between one and two intended); three data quality assessments in Kenya (between two and five intended)

Quality improvement collaboratives

- Creation of facility quality improvement teams of 3–12 people to discuss quality improvement projects and follow plan–do–study–act cycles with quality improvement coaches
- Tracking of three quality improvement indicators focused on neonatal mortality among preterm infants: gestational age assessment, antenatal corticosteroid provision, and uptake of kangaroo care
- Establishment of country-specific quality improvement collaboratives with learning sessions to discuss quality improvement indicators and change ideas
- Intervention synergy: change ideas for quality improvement generated from PRONTO simulations; quality improvement indicators informed by mSCC and maternity register

Target personnel: maternity ward and newborn care providers, facility leadership
Frequency: quality improvement facility meetings every 2 weeks and five learning sessions per country
Fidelity: five learning sessions in each country (between three and six sessions intended)

Modified Safe Childbirth Checklist

- Adaption of the WHO Safe Childbirth Checklist to focus on identification of preterm labour and management of preterm birth*
- Addition of a new pause point before admission to effectively identify preterm labour and candidates for antenatal corticosteroids or early referral
- Alignment with national guidelines and stakeholder priorities confirmed by study teams
- Intervention synergy: mSCC used during quality improvement and PRONTO activities to reinforce uptake of evidence-based practices, indicator tracking, and data use for clinical decision making

Target personnel: maternity ward and newborn care providers
Frequency: 1–2 h per month per facility (about 20 h per year), plus additional reinforcement during quality improvement and PRONTO activities (intervention sites only)
Fidelity: no specific measures of fidelity other than country-specific modification, initial training, and provision of paper checklists

PRONTO simulation and team training

- Simulation and team training that included standard basic emergency obstetric and newborn care content and emphasised prematurity-related intrapartum and immediate postnatal care practices*
- Training of PRONTO mentors who provided bedside mentoring and knowledge reviews
- Simulations and team training activities (Kenya: 4 consecutive days every 5–6 weeks; Uganda: 2 consecutive days every 6–8 weeks)
- Intervention synergy: integration of mSCC into simulations; attendance of PRONTO mentors at quality improvement sessions

Target personnel: mentees: maternity ward and newborn care providers, quality improvement team members; mentors: five nurses in Kenya, and two nurses and eight physicians in Uganda
Frequency: curriculum designed for 58 h of PRONTO activities in both countries
Fidelity: seven PRONTO trainings (five intended) plus four additional bedside mentorship visits in Uganda; 12 weeks of in-situ training and mentorship per facility in Kenya (12 weeks intended)

mSCC=modified WHO Safe Childbirth Checklist. *Including accurate gestational age assessment, use of magnesium sulphate and antenatal corticosteroids, immediate skin to skin and breastfeeding, newborn resuscitation, and preterm feeding guidelines.

Table S1. Effect (Odds Ratio, OR) of PTBi intervention on neonatal and maternal outcomes in term births (excluding the primary PTBi cohort and gestational age less than 37 completed weels) across the two countries, aggregated and separately.

	Both countries aggregate				Kenya			Uganda		
	Control	Intervention	OR (95% CI)	Interaction p-value	Control	Intervention	OR (95% CI)	Control	Intervention	OR (95 % CI)
Stillbirth + Predischarge newborn mortality (Combined)	594/14,815	243/10,188	0.77 (0.56, 1.05)	**0.006**	163/7,251	89/5,417	0.99 (0.77, 1.29)	431/7,564	154/4,771	**0.59 (0.45, 0.79)**
Stillbirth	462/14,815	177/10,188	0.74 (0.54, 1.00)	**0.009**	116/7,251	62/5,417	1.02 (0.75, 1.38)	346/7,564	115/4,771	**0.55 (0.37, 0.81)**
Predischarge newborn mortality	132/14,353	66/10,011	**0.83 (0.70, 0.97)**	0.349	47/7,135	27/5,355	0.95 (0.61, 1.47)	85/7,218	39/4,656	**0.77 (0.75, 0.79)**
*Predischarge maternal mortality**	17/11,879	12/7,405	1.19 (0.79, 1.82)	–	–	–	–	–	–	–

*Three pairs of clusters (6 facilities) were not included in this model because there were no maternal deaths in either the control or the intervention facilities. Because of small number of maternal deaths in the overall models, there was little power to investigate interaction by country.

Table S2. Proportions excluded versus included within each arm by study characteristics.

	Control		Intervention		Total	Control		Intervention		Total
	n	%*	n	%*		n	%*	n	%*	
	Livebirth					Stillbirth				
Excluded	10,103	38	8,190	41	18,293	485	40	268	47	753

Included	16,468	62	11,947	59	28,415	719	60	308	53	1,027
Total	26,571		20,137		46,708	1,204		576		1,780
	Alive at discharge					Predischarge newborn mortality				
Excluded	9,959	38	8,110	41	18,069	144	40	80	38	224
Included	16,252	62	11,816	59	28,068	216	60	131	62	347
Total	26,211		19,926		46,137	360		211		571
	Female					Male				
Excluded	5,116	38	3,992	41	9,108	5233	38	4281	41	9,514
Included	8,261	62	5,819	59	14,080	8661	62	6228	59	14,889
Total	13,377		9,811		23,188	13,894		10509		24,403
	Normal					LBW				
Excluded	9,540	38	7,533	41	17,073	812	38	709	40	1,521
Included	15,611	62	10,980	59	26,591	1347	62	1067	60	2,414
Total	25,151		18,513		43,664	2,159		1776		3,935
	Term					Preterm				
Excluded	9,514	38	7,458	41	16,972	1074	36	1000	39	2,074
Included	15,315	62	10,714	59	26,029	1872	64	1541	61	3,413
Total	24,829		18,172		43,001	2,946		2541		5,487
	APGAR score at 5 minutes >= 7					APGAR score at 5 minutes <7				
Excluded	8,216	40	7,798	41	16,014	322	28	243	36	565
Included	12,465	60	11,447	59	23,912	848	72	439	64	1,287
Total	20,681		19,245		39,926	1,170		682		1,852

	Maternal age 13 – 17 years					Maternal age 18 – 35 years				
Excluded	769	37	645	37	1,414	9107	38	7333	41	16,440
Included	1,297	63	1,085	63	2,382	14860	62	10488	59	25,348
Total	2,066		1,730		3,796	23,967		17821		41,788
	Maternal age 36 – 53 years									
Excluded	656	42	455	43	1,111					
Included	919	58	601	57	1,520					
Total	1,575		1,056		2,631					
	Vaginal delivery					Cesarean section				
Excluded	8,246	38	7,220	41	15,466	2157	38	1139	40	3,296
Included	13,449	62	10,474	59	23,923	3499	62	1724	60	5,223
Total	21,695		17,694		39,389	5,656		2863		8,519
	Singletons					Multiples				
Excluded	10,047	38	8,101	41	18,148	541	41	357	43	898
Included	16,407	62	11,780	59	28,187	780	59	475	57	1,255
Total	26,454		19,881		46,335	1,321		832		2,153

* Column total was used as denominator.

Table S3. Effect (Odds Ratio, OR) of PTBi intervention on neonatal and maternal outcomes in all included and excluded births across the two countries, aggregated and separately. (Same models as main table 2 but the sample includes both included and excluded births for sensitivity analysis to address selection bias).

	**Both countries aggregate**	**Kenya**	**Uganda**

	OR (95% CI)	Interaction pvalue	OR (95% CI)	OR (95% CI)
Stillbirth + Predischarge newborn mortality (Combined)	1.01 (0.69, 1.47)	**0.030***	1.20 (0.77, 1.86)	**0.65 (0.43, 0.97)**
Stillbirth	0.89 (0.66, 1.20)	0.068	1.03 (0.74, 1.45)	0.63 (0.39, 1.01)
Predischarge newborn mortality	0.98 (0.63, 1.53)	**0.016***	1.32 (0.84, 2.07)	**0.72 (0.61, 0.85)**
Predischarge maternal mortality	1.05 (0.85, 1.29)	0.667	0.99 (0.66, 1.49)	1.09 (0.87, 1.37)

***** Interaction p-value for Country × Intervention, suggesting statistically significant difference in the intervention effect between the two countries. Note: models are adjusted for matched pairing of facilitiesand clustering of births within facilities.

Results related to APGAR score

Table S4. Characteristics (%, n) of APGAR Scores among all births in the two countries, aggregated and separately.

	**APGAR Score @ 5 minutes <7**			
	Control (n = 17,187)		Intervention (n = 12,255)	
	%	n	%	n
Both countries aggregate	6.4	848	3.7	439
	Control (n=8,468)		Intervention (n=6,465)	
Kenya	3.7	308	3.8	242
	Control (8,719)		Intervention (5,790)	
Uganda	11.0	540	3.6	192

The proportions of neonates with APGAR score less than 7 @5 minutes were generally lower in the intervention than in the control arm. Proportions of neonates with APGAR score less than 7 @5 minutes were generally lower in Kenya than in Uganda**Supplementary Table S4**.

The effect of the intervention goes away, when the results presented in table 2 are additionally adjusted for APGAR @ 5 minutes (overall – 0.92, 95% CI: 0.68, 1.15; Kenya – 0.80, 95% CI: 0.52, 1.21; Uganda– 1.02, 95% CI: 0.63, 1.66). APGAR was very highly associated with the outcome with an OR of 360 (95% CI: 290, 456) and intervention was statistically non significantly associated with APGAR,1.16 (95% CI: 0.78, 1.73). Following the theory of change,we hypothesize that APGAR is a potential mediator, in the causal pathway between intervention and increased survival. The intervention likely improved provider's ability to assess health status of the newborn immediately after birth. The intervention included reinforcement of key actions providers should take in the first 5 minutes of life for a newborn not spontaneously breathing at birth (e.g., immediate neonatal resuscitation). Improved initial assessment and management of the condition of the baby when the mother arrives to the facility for delivery, better identification of low APGAR score newborns and improved accuracy in reporting are some other factors that likely improved due to the intervention.

CONSORT 2010 checklist

Section/Topic	Item No	Checklist item	Reported on page No
**Title and abstract**			
	1a	Identification as a randomised trial in the title	1
	1b	Structured summary of trial design, methods, results, and conclusions (for specific guidance see CONSORT for abstracts)	2
**Introduction**			
Background and	2a	Scientific background and explanation of rationale objectives 2b Specific objectives or hypotheses	3
			3
			N/A
			5-6
			4
**Methods**			
Trial design	3a	Description of trial design (such as parallel, factorial) including allocation ratio	4
	3b	Important changes to methods after trial commencement (such as eligibility criteria), with reasons	
Participants	4a	Eligibility criteria for participants	
	4b	Settings and locations where the data were collected	
Interventions	5	The interventions for each group with sufficient details to allow replication, including how and when they were actually administered	5
Outcomes	6a	Completely defined pre-specified primary and secondary outcome measures, including how and when they were assessed	6
	6b	Any changes to trial outcomes after the trial commenced, with reasons	N/A
Sample size	7a	How sample size was determined	6
	7b	When applicable, explanation of any interim analyses and stopping guidelines	N/A
Randomisation:			4-5

Sequence generation	8a	Method used to generate the random allocation sequence	
	8b	Type of randomisation; details of any restriction (such as blocking and block size)	4-5
Allocation concealment mechanism	9	Mechanism used to implement the random allocation sequence (such as sequentially numbered containers), describing any steps taken to conceal the sequence until interventions were assigned	4-5
Implementation	10	Who generated the random allocation sequence, who enrolled participants, and who assigned participants to interventions	4-5
Blinding	11a	If done, who was blinded after assignment to interventions (for example, participants, care providers, those	N/A

		assessing outcomes) and how	
	11b	If relevant, description of the similarity of interventions	5
Statistical methods	12a	Statistical methods used to compare groups for primary and secondary outcomes	6-7
	12b	Methods for additional analyses, such as subgroup analyses and adjusted analyses	7
**Results**			
Participant flow (a diagram is strongly recommended)	13a	For each group, the numbers of participants who were randomly assigned, received intended treatment, and were analysed for the primary outcome	7, Figure 1
	13b	For each group, losses and exclusions after randomisation, together with reasons	7, Figure 1
Recruitment	14a	Dates defining the periods of recruitment and follow-up	4, Supplement Figure 1
	14b	Why the trial ended or was stopped	Supplement Figure 1
Baseline data	15	A table showing baseline demographic and clinical characteristics for each group	Table 1
Numbers analysed	16	For each group, number of participants (denominator) included in each analysis and whether the analysis was by original assigned groups	7, Tables 1-3
Outcomes and estimation	17a	For each primary and secondary outcome, results for each group, and the estimated effect size and its precision (such as 95% confidence interval)	7-9
	17b	For binary outcomes, presentation of both absolute and relative effect sizes is recommended	N/A 8-9
Ancillary analyses	18	Results of any other analyses performed, including subgroup analyses and adjusted analyses, distinguishing pre-specified from exploratory	

Harms	19	All important harms or unintended effects in each group (for specific guidance see CONSORT for harms)	N/A
**Discussion**			
Limitations	20	Trial limitations, addressing sources of potential bias, imprecision, and, if relevant, multiplicity of analyses	10-11
Generalisability	21	Generalisability (external validity, applicability) of the trial findings	9-11
Interpretation	22	Interpretation consistent with results, balancing benefits and harms, and considering other relevant evidence	9-11
**Other information**			
Registration	23	Registration number and name of trial registry	7
Protocol	24	Where the full trial protocol can be accessed, if available	Citation #5
Funding	25	Sources of funding and other support (such as supply of drugs), role of funders	12
